# Role of novel type I interferon epsilon in mucosal immunity

**DOI:** 10.1186/1742-4690-9-S2-P192

**Published:** 2012-09-13

**Authors:** Y Xi, SL Day, RJ Jackson, C Ranasinghe

**Affiliations:** 1 John Curtin School of Medical Research, Canberra, Australia

## Background

Newly discovered type I interferon-epsilon (IFN-ε) is found to be constitutively expressed in mucosal tissues, i.e lung, reproductive tissue and intestine. Our previous studies have postulated that IFN-ε could play a role in modulating mucosal immunity. As HIV is a disease of the mucosae, we further evaluated the immuno-biology of IFN-ε in the mucosae and tested whether IFN-ε could be used as a mucosal adjuvant to enhance HIV-specific immunity.

## Methods

Poxvirus (Vaccinia Virus and Fowl poxvirus) co-expressing HIV-1 gag/pol and interferon epsion (VV-HIV-IFN-ε or FPV-HIV-IFN-ε) were used in this study to evaluate immuno-biology and adjuvant activity of IFN-ε.

## Results

Firstly, VV-HIV-IFN-ε was utilized to study the immuno-biology of IFN-ε compared to IFN-α4 or IFN-β. Following intranasal (i.n.) VV-HIV-IFN-ε infection, a rapid VV clearance in lung was induced that correlated with 1) an elevated lung VV-specific CD8+CD107a+IFN-γ+, 2) up-regulated activation markers CD69/CD103 on CD8 T cells, 3) enhanced lymphocyte recruitment to lung alveoli with reduced inflammation and 4) heightened functional/cytotoxic CD8+CD4+ T cell subset (CD3hiCCR7hiCD62Llo) in lung lymph nodes. These responses were different to that observed following i.n. VV-HA-IFN-α4 or VV-HA-IFN-β infections. Secondly, intranasal/intramuscular (i.n./i.m.) heterologous prime-boost immunization (FPV-HIV-IFN-ε/VV-HIV-IFN-ε) was used to evaluated adjuvant activity of IFN-ε. Data indicated that IFN-ε induced elevated HIV-specific effector but not memory CD8 T cells responses in spleen, genito-rectal nodes and Peyer’s patch compared to the control (i.n. FPV-HIV/i.m. VV-HIV). Interestingly, unlike IFN-β and IFN-α4, IFN-ε uniquely induce elevated frequency of α4β7 and CCR9 expressing HIV-specific CD8 T cells in gut mucosae.

## Conclusion

In conclusion, our data indicated that 1) IFN-ε can induced excellent T cell response in the mucosae especially lung and gut, and 2) rather than an vaccine adjuvant IFN-ε has the potential to be used as an anti-microbicide to prevent or reduced mucosal infection such as TB or HIV.

